# Prevalence of polycystic ovary syndrome and determinants of knowledge among women in the eastern province Saudi Arabia

**DOI:** 10.3389/frph.2026.1780568

**Published:** 2026-04-01

**Authors:** Deema AL Shawan, Maryam Alsaad, Bayan Alhumaid, Fatimah Shabib, Salma Almulla, Danah AlThukair, Haneen Alfawwaz

**Affiliations:** College of Public Health, Kingdom of Saudi Arabia, Imam Abdulrahman bin Faisal University, Dammam, Saudi Arabia

**Keywords:** awareness, knowledge level, polycystic ovary syndrome, prevalence, Saudi Arabia, women's health, PCOS

## Abstract

**Background:**

Polycystic Ovary Syndrome is a common condition among women of reproductive age, yet many cases remain undiagnosed. While several studies have assessed awareness in Saudi Arabia, data from the Eastern Province are limited.

**Objectives:**

To measure the prevalence of Polycystic Ovary Syndrome among women aged 18–45 in the Eastern Province, evaluate their knowledge of the condition, and examine demographic factors associated with prevalence and knowledge levels.

**Methods:**

A cross-sectional online survey was administered to 408 women recruited. The questionnaire collected data on sociodemographic characteristics, knowledge levels, and symptom-based indicators related to Polycystic Ovary Syndrome. Data were analyzed using descriptive statistics and multiple linear regression analysis.

**Results:**

Symptom-based prevalence was 19.9 per cent, and 16.7 per cent reported a formal diagnosis. Awareness was high (83.1 percent), and 61.5 percent showed good knowledge. Demographic factors were not significantly associated with prevalence. Knowledge was significantly associated with age, medical or health-related background, and income (*p* > 0.05).

**Conclusion:**

Although awareness is relatively high, knowledge gaps remain, especially among women without health-related backgrounds. Tailored educational and early detection efforts are needed to improve understanding and support timely diagnosis and management.

## Introduction

Despite being one of the most widespread hormonal disorders among women, Polycystic Ovary Syndrome (PCOS) often hides in plain sight, with almost 70% of affected women worldwide remaining undiagnosed. According to the World Health Organization, PCOS is estimated to affect between 6% and 13% of women of reproductive age (1). Recent global epidemiological evidence highlights a steady rise in the prevalence of polycystic ovary syndrome worldwide. A comprehensive age-stratified analysis covering the period from 1990 to 2021 revealed a consistent upward trend in PCOS prevalence over time. In 2021, the highest incidence was observed among adolescents aged 10–14 years, with progressively lower incidence rates reported in older age groups (2). Beyond its impact on reproductive health, PCOS is associated with significant long-term metabolic and psychological complications, underscoring its status as a substantial yet under-recognized public health concern (1, 3, 4).

Despite the high prevalence of PCOS, research investigating women's knowledge of the condition and its association with demographic factors remains limited. Within Saudi Arabia, only a few studies have addressed this topic. Much of the existing literature has focused primarily on estimating prevalence within specific populations, such as university students. For example, a study conducted at a university in Riyadh reported a PCOS prevalence of 16% (3). Other studies have been conducted in different cities or regions of the Kingdom. For instance, a study reported a prevalence of 31.8% among women in the Western Region of Saudi Arabia (4). Furthermore, researchers conducted a cross-sectional study in Al Madinah City among women aged 18–45 years and found that 32.5% of participants were diagnosed with PCOS. The study identified substantial variation in prevalence across demographic characteristics, including age group, marital status, occupation, socioeconomic status, and educational attainment. Moreover, PCOS affected 32.6% of Saudi women and 31% of non-Saudi women, indicating an almost equal distribution between the two groups (5).

As for awareness levels, a study conducted among Saudi women in Madinah City and found that 56.7% of the 2,000 participants had a high awareness level of PCOS. The investigators also found that women with higher educational attainment, particularly those who graduated from health-related fields, demonstrated significantly greater knowledge of the condition (6). On the other hand, other studies revealed low awareness of PCOS among participants, with most demonstrating moderate to poor understanding.

PCOS is one of the most common reproductive disorders affecting women of reproductive age, as reported in regional data from Middle Eastern countries (7). These findings underscore the importance of conducting province-specific research in Saudi Arabia in order to appropriately measure local prevalence and awareness levels

Nevertheless, there remains a clear lack of research examining the prevalence and level of awareness of Polycystic Ovary Syndrome among women aged 18–45 years residing in the Eastern Province of Saudi Arabia. Geographical variations in cultural, educational, and socioeconomic factors may influence women's awareness, diagnosis, and perception of PCOS, underscoring the need for region-specific data to improve knowledge and inform targeted health awareness programs (1, 8, 9). To address this gap, the present study aimed to determine the prevalence of PCOS among women, assess their knowledge of the condition, and examine associations between demographic factors and the syndrome. The findings are intended to provide valuable insights to guide public health policies, healthcare services, and relevant awareness initiatives in Saudi Arabia. Furthermore, several lifestyle variables increase the likelihood of developing PCOS, including being overweight, physically inactive, having a poor diet, and having a family history of the disease (10).

This focus is particularly important in the Saudi and Middle Eastern context due to the high prevalence of metabolic risk factors and the potential hereditary influences associated with the high rate of consanguineous marriages (11). Additionally, because reproductive health is often considered a sensitive and less openly discussed topic in the region, limited access to accurate information may contribute to misconceptions, reduced awareness, and delayed help-seeking behavior (12).

Therefore, improving the health of women in Saudi Arabia requires an understanding of the population's level of awareness regarding PCOS. As a result, measuring awareness levels will help to identify knowledge gaps and develop educational initiatives that support early diagnosis and effective treatment of this illness.

## Materials and methods

A descriptive cross-sectional study design was used to assess the prevalence of PCOS and the level of knowledge about the condition among Saudi women. A non-random convenience sampling method was employed. Volunteers were recruited to participate in this study were collected via an online questionnaire distributed via various social media platforms, including X, LinkedIn, and WhatsApp.

The inclusion criteria were women aged 18–45 years who currently reside in the Eastern Province of Saudi Arabia and were willing to participate in the survey. More specifically, data were collected from participants residing in the province's main cities, namely Dammam, Khobar, Al-Ahsa, Qatif, and Jubail.. Regarding the age group, participants were analysed using the broader 18–45-year age range rather than the alternative age categories. These categories were commonly used in previous research on PCOS, as they represent the reproductive period and are most relevant for assessing prevalence, symptoms, and knowledge of the condition (13).

The sample size was calculated based on a population size of approximately 822,000 women in the Eastern Province, using a 95% confidence level, a 5% margin of error, and a response distribution of 50%. The standard sample size formula $n=Z^{2}P(1-P)/d^{2}$ was applied, where Z=1.96 for a 95% confidence interval, P=0.5, and d=0.05. This calculation resulted in a minimum required sample size of 384 participants for the study. Although the minimum required sample size was 384 respondents, a total of 408 participants completed the survey, and all 408 responses were considered in the analysis.

Data were collected using a pre-tested, self-administered, structured questionnaire adapted from the validated Polycystic Ovary Syndrome Screening Questionnaire (13). The study questionnaire was structured into three main sections. The first section assessed participants’ demographic characteristics and consisted of eight multiple-choice questions. Subsequently, the second section evaluated the prevalence of PCOS which included a twelve dichotomous (yes/no) questions. For this purpose, a binary scoring system was applied, whereby each “yes”= a score (1), while each “no”= a score (0); consequently, the total score ranged from 0 to 12. Based on this scoring system, participants with scores from 0 to 6 were classified as having no PCOS, whereas those with scores from 7 to 12 were classified as potentially having PCOS.

Finally, the third section assessed participants’ knowledge of PCOS and comprised fourteen multiple-choice questions. In this section, each correct response was awarded one point, while incorrect responses received zero points, yielding a total knowledge score ranging from 0 to 14. To further classify knowledge levels, two classification approaches were applied: in the first-level classification, scores of 0–4 indicated low knowledge, 5–9 indicated moderate knowledge, and 10–14 indicated high knowledge; additionally, in the second-level classification, scores of 0–6 were classified as poor knowledge, while scores of 7–14 were classified as good knowledge (13).

The questionnaire was translated into Arabic and validated through back translation to ensure accuracy and equivalence. Moreover, the questionnaire underwent a two-stage validation process. First, the questionnaire was reviewed for face and content validity by four experts in the field to ensure that all items are clear, relevant and able to measure the intended concepts. Following this, a pilot study was conducted with 37 participants, representing approximately 10% of the target sample size (*n* = 384). Cronbach's alpha was used to assess the internal consistency of the knowledge section, and the coefficient was 0.91. This number validates the instrument's appropriateness for the full-scale investigation and shows outstanding dependability.

All responses were automatically recorded through the QuestionPro platform and analyzed using SPSS Statistics V26. Inferential analyses were then conducted using multiple linear regression assessing the relationship between demographic variables and PCOS prevalence, as well as to assess the association between demographic factors and participants’ level of knowledge regarding PCOS.

The ethical approval was secured from the Institutional Review Board (IRB) of Imam Abdulrahman bin Faisal University (IAU) under number IRB-UGS-2025-03-0031. Statistical analysis was performed using the IBM SPSS Statistics Version 23.0. Descriptive statistics, including frequencies, percentage, means, and standard deviations, were calculated for all items within the demographic characteristics, PCOS prevalence, and PCOS knowledge section of the questionnaire, responses. This provided a summary of the distribution of responses for all variables. For inferential analysis, multiple linear regression was conducted to assess the relationship between demographic variables and PCOS prevalence, and knowledge level. A *p*-value of less than 0.05 was considered the threshold for statistical significance.

## Results

### Demographic characteristics of the study participants

Table 1 presents the demographic characteristics of the 408 participants. The largest age group was 21–29 years (34.8%), followed by participants aged 30–39 years (23.8%) and those aged 40–45 years (22.1%). Participants aged 18–20 years represented 19.4% of the sample. The vast majority were Saudi citizens (96.8%). Regarding current residence, most participants lived in Al-Ahsa (43.4%), followed by Dammam (30.6%), Qatif (14.2%), and Khobar (8.1%), while smaller proportions resided in Jubail (2%) or other areas (1.7%). In terms of educational attainment, nearly half of the participants held a university degree (49.5%), while 42.8% had completed high school; only 3.9% held a diploma and 3.7% had pursued postgraduate studies. With respect to marital status, 52.8% were married, 42.4% were single, and smaller percentages were divorced (2.9%) or widowed (1.7%). A majority of participants (59.8%) reported having knowledge of health or medicine, whereas 40.2% reported no such knowledge. Monthly household income was distributed across categories, with 34.3% earning between 5,000 and 10,000 SAR, 31.1% earning more than 10,000 SAR, and 30.6% earning less than 5,000 SAR; an additional 3.9% reported other income levels.

**Table 1:** Demographic characteristics of study participants.

Demographic factors	Frequency	Percentage
Age group		
18–20 years	79	19.4%
21–29 years	142	34.8%
30–39 years	97	23.8%
40–45 years	90	22.1%
Total	408	100%
Saudi citizen		
Yes	395	96.8%
No	13	3.2%
Total	408	100%
Current residence		
Dammam	125	30.6%
Khobar	33	8.1%
Al-ahsa	177	43.4%
Qatif	58	14.2%
Jubail	8	2%
Other	7	1.7%
Total	408	100%
Highest education level		
High school	175	42.8%
Diploma	16	3.9%
University	202	49.5%
Post-graduate studies	15	3.7%
Total	408	100%
Marital status		
Single	173	42.4%
Married	216	52.8%
Divorced	12	2.9%
Widowed	7	1.7%
Total	408	100%
Medical or health background		
Yes, I have knowledge of health or medicine	244	59.8%
No, I have no knowledge	164	40.2%
Total	408	100%
Monthly household income		
Less than 5.000 SAR	125	30.6%
5.000 to 10.000 SAR	140	34.3%
More than 10.000 SAR	127	31.1%
Other	16	3.9%
Total	408	100%

### Assessment of participants’ knowledge of PCOS

Table 2 illustrates the level of knowledge about PCOS among the 408 participants. First, 83.1% of respondents had heard of the term “Polycystic Ovary Syndrome,” while 65.4% were familiar with androgens such as testosterone. Furthermore, 29.4% were aware that PCOS involves an increased level of androgens, and 27.2% recognized that women with PCOS often have multiple small cysts in their ovaries.

**Table 2:** Knowledge of polycystic ovary syndrome Among Saudi women in the eastern province, Saudi Arabia.

Knowledge about polycystic ovary syndrome (PCOS)	Frequency	Percentage
Have you heard of the term “Polycystic Ovary Syndrome (PCOS)”?		
Yes	339	83.1%
No	48	11.8%
I do not know	21	5.1%
Total	408	100%
Have you heard of the androgen hormone (such as “testosterone”)?		
Yes	267	65.4%
No	107	26.2%
I do not know	34	8.3%
Total	408	100%
In (PCOS), there is an increased level of androgens.		
Yes	120	29.4%
No	43	10.5%
I do not know	245	60%
Total	408	100%
Patients with (PCOS) have multiple small cysts in the ovaries.		
Yes	111	27.2%
No	31	7.6%
I do not know	266	65.2%
Total	408	100%
Obesity may cause (PCOS).		
Yes	260	63.7%
No	21	5.1%
I do not know	127	31.1%
Total	408	100%
Prediabetes may lead to (PCOS).		
Yes	153	37.5%
No	27	6.6%
I do not know	228	55.9%
Total	408	100%
Irregular or absent menstrual periods are a symptom of (PCOS).		
Yes	300	73.5%
No	12	2.9%
I do not know	96	23.5%
Total	408	100%
Abnormal hair growth in various parts of the body is a symptom of (PCOS).		
Yes	247	60.5%
No	25	6.1%
I do not know	136	33.3%
Total	408	100%
Severe acne during the menstrual cycle is a symptom of (PCOS).		
Yes	169	41.4%
No	42	10.3%
I do not know	197	48.3%
Total	408	100%
Hair loss from the scalp more than usual is a symptom of (PCOS).		
Yes	174	42.6%
No	28	6.9%
I do not know	206	50.5%
Total	408	100%
(PCOS) can be diagnosed with a transvaginal ultrasound.		
Yes	182	44.6%
No	21	5.1%
I do not know	205	50.2%
Total	408	100%
A blood test can be used to diagnose (PCOS).		
Yes	133	32.6%
No	58	14.2%
I do not know	217	53.2%
Total	408	100%
Which of the following side effects can be caused by (PCOS)?		
Only heart diseases	0	0%
Only infertility or reduce fertility	87	21.3%
Only anxiety and depression	40	9.8%
Heart diseases—Anxiety and depression—Infertility or reduce fertility	20	4.9%
Heart diseases—Anxiety and depression	1	0.2%
Heart diseases—Infertility or reduce fertility	5	1.2%
Anxiety and depression—Infertility or reduce fertility	162	39.7%
I don't know	93	22.8%
Total	408	100%
Which of the following treatments can be used for (PCOS)?		
Hormonal therapy (contraceptives)	58	14.2%
Antidiabetic medications (such as metformin)	12	2.9%
other medications (such as clomiphene, letrozole)	6	1.5%
Surgery to remove ovarian cysts	30	7.4%
Hormonal therapy- Antidiabetic—other medications—Surgery to remove ovarian cysts	11	2.7%
Hormonal—Antidiabetic—other medication (such as clomiphene, letrozole)	9	2.2%
Hormonal -other Hormonal-other medication—Surgery	8	2%
Hormonal—Antidiabetic- Surgery	20	4.9%
Hormonal—Antidiabetic	23	5.6%
Hormonal—other medications (such as clomiphene, letrozole)	8	2%
Hormonal—Surgery	57	14%
Antidiabetic—other medications (such as clomiphene, letrozole)	4	1%
Antidiabetic- Surgery	4	1%
Antidiabetic—other medications (such as clomiphene, letrozole) -Surgery	2	0.5%
Other medication—Surgery	7	1.7%
I don't know	153	37.5%
Total	408	100%
Overall Knowledge levels		
Low knowledge: 0–4	108	26.4%
Moderate knowledge: 5–9	157	38.5%
high knowledge:10–14	143	35%
Total	408	100%

When asked about contributing factors, 63.7% believed obesity might cause PCOS, and 37.5% acknowledged that pre-diabetes may also lead to the condition. Moreover, the majority (73.5%) understood that irregular or absent menstrual periods are symptoms of PCOS, while 60.5% associated abnormal hair growth, and 41.4% identified severe acne during the menstrual cycle as a related symptom.

Regarding diagnostic knowledge, 42.6% believed scalp hair loss to be a symptom, and 44.6% knew that PCOS could be diagnosed via transvaginal ultrasound. Additionally, 32.6% were aware that a blood test can be used for diagnosis. As for the side effects, 39.7% believed PCOS might lead to anxiety, depression, infertility, or reduced fertility. Additionally, most participants showed limited knowledge of treatment options, as 37.5% selected “I don’t know” when asked about available treatments.

Finally, most of the participants had knowledge about PCOS, as shown by the second-level classification, in which 61.5% were categorized as having good knowledge (scores 7–14), while 38.5% had poor knowledge (scores 0–6). Similarly, based on the first classification method, 35% of participants demonstrated high knowledge (scores 10–14), 38.5% had moderate knowledge (scores 5–9), and 26.4% showed low knowledge (scores 0–4).

### Prevalence of PCOS

Overall, 17.9% of participants reported a family history of PCOS, while 16.7% had received an official diagnosis. In terms of menstrual irregularities, 23.5% experienced very heavy bleeding, and 29.2% reported prolonged menstrual cycles. Additionally, 12.7% reported a complete absence of menstruation, and 29.9% experienced partial absence.

Acne during the menstrual cycle was reported by 45.6% of participants. Hair-related symptoms were also prevalent: 45.1% experienced scalp hair loss, and 38.5% reported abnormal hair growth on different areas of the body. Moreover, 44.1% of respondents noted skin discoloration or darkened patches, while only 3.7% indicated having diabetes.

Based on the self-reported symptoms, 81 participants (19.9%) met the criteria for PCOS, reporting prevalence scores between 7 and 12. In contrast, 327 participants (80.1%) did not meet the criteria for PCOS (Table 3).

**Table 3:** Prevalence of PCOS among participants, in the eastern province, Saudi Arabia.

PCOS indicator	Frequency	Percentage
Do you have a history of PCOS in your mother or sister?		
Yes	73	17.9%
No	335	82.1%
Total	408	100%
Do you have very heavy periods (more than 1 pad per hour)?		
Yes	96	23.5%
No	312	76.5%
Total	408	100%
Do you have prolonged periods (more than 7 days)?		
Yes	119	29.2%
No	289	70.8%
Total	408	100%
Have you been diagnosed with Polycystic Ovary Syndrome (PCOS)?		
Yes	68	16.7%
No	340	83.3%
Total	408	100%
Do you have a complete absence of a menstrual cycle (not at all)?		
Yes	52	12.7%
No	356	87.3%
Total	408	100%
Do you have a partial absence of a menstrual cycle (not after 28 days)?		
Yes	122	29.9%
No	286	70.1%
Total	408	100%
Do you have acne problems during your menstrual cycle?		
Yes	186	45.6%
No	222	54.4%
Total	408	100%
Do you have an unusual amount of hair loss from your scalp?		
Yes	184	45.1%
No	224	54.9%
Total	408	100%
Do you have an unusual amount of hair growth at different parts of your body (upper lip, chin, abdomen, breast, thighs, etc.)?		
Yes	157	38.5%
No	251	61.5%
Total	408	100%
Do you have discoloration or dark-colored patches on your skin?		
Yes	180	44.1%
No	228	55.9%
Total	408	100%
Do you have continuous abnormal weight gain?		
Yes	111	27.2%
No	297	72.8%
Total	408	100%
Do you have diabetes?		
Yes	15	3.7%
No	393	96.3%
Total	408	100%
Prevalence of PCOS		
Absence of PCOS: 0–6 prevalence Score	327	80.1%
Presence of PCOS: 7–12 prevalence Score	81	19.9%
Total	408	100%

### The associations between demographic factors and PCOS prevalence

None of the demographic variables were significantly associated with PCOS prevalence scores. Age group (*p* = 0.146), Saudi citizenship (*p* = 0.060), current residence (*p* = 0.857), educational level (*p* = 0.677), marital status (*p* = 0.574), medical or health background (*p* = 0.646), and monthly household income (*p* = 0.126) did not demonstrate significant predictive value in the model (Table 4).

**Table 4:** The association between demographic factors and PCOS prevalence.

Predictor variable	Unstandardized coefficient (B)	*P*-value
Age group	−0.226	0.146
Saudi citizenship	1.293	0.06
Current residence	−0.018	0.857
Highest education level	0.071	0.677
Marital status	−1.138	0.574
Medical or health background	0.073	0.646
Monthly household income (economic status)	−0.212	0.126

### The association between demographic factors and the knowledge score PCOS

Table 5 presents the association between demographic characteristics and knowledge levels. Age group showed a significant negative association with knowledge (B = –0.651, *p* = 0.006), indicating that younger participants had higher knowledge scores. Having a medical or health-related background was also a significant predictor (B = –0.812, *p* < 0.001), suggesting higher knowledge among those with such backgrounds. Monthly household income demonstrated a modest but significant positive association (B = 0.417, *p* = 0.048). Other demographic variables, including citizenship, residence, education level, and marital status, were not statistically significant predictors of knowledge level.

**Table 5:** The association between demographic factors and knowledge level.

Predictor variable	Unstandardized coefficient (B)	*P*-value
Age group	−0.651	0.006
Saudi citizenship	−0.349	0.737
Current residence	−0.051	0.732
Highest education level	0.25	0.330
Marital status	−0.55	0.139
Medical or health background	−0.812	<0.001
Monthly household income (economic status)	0.417	0.048

## Discussion

Globally, PCOS affects approximately 6%–13% of women of reproductive age, with up to 70% of cases remaining undiagnosed (1). Similarly, our findings revealed that 19.9% of participants met the prevalence score criteria, while a slightly lower proportion, 16.7%, had a formal PCOS diagnosis. These rates offer valuable insight into the burden of PCOS within this region. On the other hand, the prevalence in other regions was much higher. For example, a study conducted in Madinah reported a prevalence of 32.5%, while another in the Western region reported 31.8% (4, 6). In Qatar, the prevalence ranged from 12.1% to 20%, depending on the diagnostic criteria used (14). Furthermore, a study conducted on Saudi women indicated that despite having the symptoms, 42.1% delayed seeking medical care for 1–4 years, which may contribute to delayed management of PCOS (15). This suggests that PCOS prevalence may vary across regions, highlighting the importance of local studies and early screening.

As for PCOS knowledge, 83.1% of participants were aware of the condition, and 61.5% demonstrated a good level of knowledge. These findings are consistent with a previous study that reported a 56.7% awareness rate among Saudi females (8). However, more recent studies in the Kingdom have shown contrasting results. For example, Bukhari et al. and Radwan et al. found generally low levels of PCOS awareness, with most participants exhibiting only moderate to poor understanding (4, 5). Therefore, our study indicates a likely growing awareness of PCOS among women in Saudi Arabia, perhaps resulting from enhanced access to information and healthcare programs.

The findings of the current study partially align with those reported by a study that examined the association between demographic factors and PCOS knowledge in Hail City, Saudi Arabia. Both studies identified age and having a medical or health-related background as significant predictors of knowledge levels. Additionally, in both studies, younger participants demonstrated higher knowledge scores, whereas other researchers reported that individuals under 30 years had significantly higher knowledge than those over 30 (16).

This research provides valuable insights into the prevalence and awareness of PCOS among women in Saudi Arabia's Eastern Province. The findings highlight the necessity of ongoing efforts to improve understanding and awareness of PCOS, particularly among specific demographic groups, as the syndrome can increase the risks of developing cardiovascular disease, pregnancy-related complications, and psychological problems (17). Furthermore, 17.9% of the participants have a history of PCOS in their family, which increases the importance of continuous education and providing updated information for the future generation, as they might be at a higher risk due to family history. Previous research has highlighted that genetic factors should be considered when monitoring for PCOS prevalence (18).

Future research could examine the sources of information that women in this region rely on and assess the effectiveness of various educational strategies to increase PCOS awareness and promote early detection and treatment. The wide use of social media could be a tool to educate the community and reach out to different age groups by providing an official source of information that can be updated and controlled (19). Furthermore, educational approach needs to be accessible and relevant to the concerns of different age groups (20).

This study has several limitations that should be acknowledged. First, it relied on self-reported screening measures, which may have resulted in undetected or inaccurately reported cases of PCOS due to recall bias. Second, the use of convenience sampling limits the representativeness of the sample, and the findings may not be fully generalizable to the broader population. Lastly, this study was limited to the main cities of the Eastern Province and did not include rural or smaller communities. However, approximately three-fifths of the Saudi population resides in major urban areas, which typically have well-developed infrastructure, integrated transportation networks, and access to essential services, making the findings broadly relevant to the majority of the province's population (21).

## Conclusion

The findings of this study shed light on the prevalence and knowledge of PCOS among women in the Eastern Province of Saudi Arabia. Although almost one-fifth of participants met the symptom-based criteria for the condition, and general awareness was relatively high, clear knowledge gaps were identified, particularly among women without a medical or health-related background and those with lower household income. While demographic factors were not significantly associated with prevalence, the variations in knowledge highlight the need for more focused educational and preventive efforts. Strengthening understanding of PCOS is essential to improving early detection, supporting timely diagnosis, and enhancing women's reproductive health outcomes.

Given the marked differences in diagnosis and level of knowledge among the women participants, it is strongly recommended that standardised national guidelines for the diagnosis and treatment of polycystic ovary syndrome be developed and implemented to ensure standardised diagnosis and long-term personalised treatment for affected women (22). Furthermore, it is essential to strengthen the role of primary healthcare services in raising awareness, conducting early screenings, and providing prompt referrals, as this improves access to care and ensures continuity of treatment. The study showed that BMI was the factor most strongly associated with the prevalence of the syndrome. Therefore, it recommends encouraging women to follow a healthy diet, exercise regularly, get enough sleep, and control their weight. This has been shown to significantly improve metabolic, hormonal, and reproductive outcomes in women with polycystic ovary syndrome (23). Additionally, greater emphasis on early screening is recommended to identify signs of PCOS, including acne, irregular menstrual cycles, and hirsutism (24). Given that only 16.7% of cases were formally diagnosed in this study, the Ministry of Health, in collaboration with local healthcare authorities, primary care clinics, and schools in the Eastern Province, should implement a routine periodic screening program for women starting at age 16. Special attention should be given to those with a family history of PCOS to facilitate early detection and timely intervention.

## Data Availability

The raw data supporting the conclusions of this article will be made available by the authors, without undue reservation.
